# Linear B-Cell Epitope Prediction for In Silico Vaccine Design: A Performance Review of Methods Available via Command-Line Interface

**DOI:** 10.3390/ijms22063210

**Published:** 2021-03-22

**Authors:** Kosmas A. Galanis, Katerina C. Nastou, Nikos C. Papandreou, Georgios N. Petichakis, Diomidis G. Pigis, Vassiliki A. Iconomidou

**Affiliations:** Section of Cell Biology and Biophysics, Department of Biology, School of Sciences, National and Kapodistrian University of Athens, 15701 Athens, Greece; kosmasgal@gmail.com (K.A.G.); katnastou@biol.uoa.gr (K.C.N.); npapand@biol.uoa.gr (N.C.P.); g.petihakis@gmail.com (G.N.P.); dpigis@gmail.com (D.G.P.)

**Keywords:** B-cell epitope, linear epitope, consensus prediction method, immunotherapy, vaccine design

## Abstract

Linear B-cell epitope prediction research has received a steadily growing interest ever since the first method was developed in 1981. B-cell epitope identification with the help of an accurate prediction method can lead to an overall faster and cheaper vaccine design process, a crucial necessity in the COVID-19 era. Consequently, several B-cell epitope prediction methods have been developed over the past few decades, but without significant success. In this study, we review the current performance and methodology of some of the most widely used linear B-cell epitope predictors which are available via a command-line interface, namely, BcePred, BepiPred, ABCpred, COBEpro, SVMTriP, LBtope, and LBEEP. Additionally, we attempted to remedy performance issues of the individual methods by developing a consensus classifier, which combines the separate predictions of these methods into a single output, accelerating the epitope-based vaccine design. While the method comparison was performed with some necessary caveats and individual methods might perform much better for specialized datasets, we hope that this update in performance can aid researchers towards the choice of a predictor, for the development of biomedical applications such as designed vaccines, diagnostic kits, immunotherapeutics, immunodiagnostic tests, antibody production, and disease diagnosis and therapy.

## 1. Introduction

B-cell epitopes are regions on the surface of an antigen, to which specific antibodies recognize and bind, triggering the immune response [1]. This interaction is at the core of the adaptive immune system, which among others is responsible for immunological memory and antigen-specific responses in vertebrates [2]. The ability to identify these binding areas in the antigen’s sequence or structure is important for the development of synthetic vaccines [3,4,5], diagnostic tests [6], and immunotherapeutics [7,8], especially in the COVID-19 era. Focus on these applications through the lens of epitope discovery has gained attention over the years, especially in regard to the safety benefits of synthetic vaccine development [9].

Generally, B-cell epitopes are divided into two categories: linear (continuous) epitopes, which consist of a linear sequence of residues; and conformational (discontinuous) epitopes, which consist of residues that are not contiguous in the primary protein sequence but are brought together by the folded protein structure [10]. Moreover, the vast majority of B-cell epitopes have been estimated to be conformational, while only a fraction are linear [11]. Nonetheless, it has been shown that many discontinuous epitopes contain several groups of continuous residues that are also contiguous in the tertiary structure of the protein [12], making the distinction between them unclear.

All aforementioned immunological applications share the need for the discovery of all possible epitopes for any given antigen, a process called “Epitope mapping”. Although epitope mapping can be carried out using several experimental techniques [13], computational methods can complement the already existing methods and possibly speed up research [14]. To address this point and tap into the ever-growing data on epitopes deposited in biological databases daily, several computational methods for predicting conformational or linear B-cell epitopes have been published over the last decades [15,16,17]. Despite the relatively small percentage of linear B-cell epitopes, most methods developed over the past few years focus on their prediction. This is mainly attributed to the requirement of an antigen’s 3D structure when predicting its conformational epitopes [18]. Thus, in this review, we will discuss solely the performance of linear B-cell epitope (BCE) predictors.

In most cases, the algorithms that predict BCEs can either be sequence-based and/or structure-based. Most predictors utilize only data derived from the protein sequence of the antigen and thus are sequence-based, while structure-based predictors utilize only an antigen’s 3D structure. Furthermore, some hybrid methods employ both approaches for better predictive performance [19,20]. Historically, initial attempts at predicting epitopes made use of a single amino acid propensity scale, assigning each amino acid a numerical value, followed by a local averaging of these values along the peptide chain. The first method, implementing this approach, was published by Hopp and Woods [21] in 1981, and it utilized Levitt’s hydrophilicity scale [22]. Aside from hydrophilicity, which was utilized again in another scale by Parker et al. [23], other amino acid properties were explored in later methods, such as antigenicity [24], flexibility [25], surface accessibility [26], and turns [27]. The next wave of predictors built upon this development, when methods like PREDITOP [28], PEOPLE [29], BEPITOPE [30], and BcePred [31], combined multiple physicochemical properties. Although these methods represented the best attempts yet at predicting epitopes, Blythe and Flower [32] demonstrated that the performance of such methods was overstated. They did a thorough assessment of 484 amino acid propensity scales in combination with information on the location of epitopes for 50 known proteins and found that even the best possible combination of scales performed only slightly better than random [32]. In their work they also correctly suggested that more advanced approaches for predicting linear B-cell epitopes needed to be developed, such as methods that employ artificial intelligence technology.

As anticipated, given the booming of available biological data, the entire next generation of methods utilized some form of machine learning models. One of the first such approaches was BepiPred [33], which combined a Hidden Markov Model (HMM) with an amino acid propensity scale. Additionally, other machine learning models were used in methods developed afterwards, including Neural Networks in ABCpred [34], a Naïve Bayes classifier in Epitopia [35], and Support Vector Machines (SVMs) in most of the recent predictors. SVM-based predictors dominated the machine learning approaches used in BCE prediction, each one differing from the other on feature selection, data set curation and SVM specific parameters (Table 1). The BCPred [36] and FBCPred [37] methods published in 2008, predict fixed linear B-cell epitopes and flexible length linear B-cell epitopes respectively, utilizing SVM models with the subsequence kernel. The AAPPred [38] method also utilizes SVM models trained on the frequency of Amino Acid Pairs (AAP), a scale first developed by Chen et al. [39]. Other notable approaches include: BayesB [40], LEPS [41], and BEOracle [42]. A new machine learning approach that was developed in 2014, called EPMLR [43], utilizes multiple linear regression for epitope classification. Another recent novel approach is the DMN-LBE [44] method, which was developed using deep maxout networks, a type of deep neural network with a different activation layer called maxout. The DRREP [45] method was published in 2016, and it also utilizes deep neural network technology to extrapolate structural features related to epitopes from protein sequences. One of the latest additions is the second version of the BepiPred method, BepiPred-2.0 [20], which was developed in 2017. This method is based on a random forest algorithm and differs from its predecessor in that it was trained only on epitope data derived from crystal structures. Another promising algorithm is iBCE-EL [46], which is an ensemble learning framework combining Extremely Randomized Tree (ERT) and Gradient Boosting (GB) classifiers. An overview of all methods is presented in Table 1 below.

Here, we review the performance of some of the most widely used linear B-cell epitope predictors currently available via a Command-Line Interface (CLI), namely BcePred [31], BepiPred [33], ABCpred [34], COBEpro [19], SVMTriP [49], LBtope [51], and LBEEP [53]. We also examine the performance of a consensus classifier combining these methods, to test whether a consensus approach can boost predictive performance. This was decided in order to attempt to remedy performance issues of the individual methods, since consensus classifiers have been previously shown to outperform constituent classifiers in some cases [55,56,57]. Finally, we compare the performance of all these classifiers and the consensus method we developed against one of the most recently published BCE predictors, BepiPred-2.0 [20]. Aside from being one of the latest developed methods, Bepipred-2.0 was also chosen because of its testing data set, which was used for our testing needs, giving us the much-needed testing methodology overlap to adequately compare newer and older methods. This review aims to give non-expert researchers an overview of available linear BCE predictors, as well as an update in their current performance and availability, which they can use to quickly locate and choose the appropriate tools for their research work. Moreover, we have created contemporary non-redundant datasets of linear BCEs that could aid both experimental researchers as well as bioinformaticians actively working in the field of algorithm development.

## 2. Materials and Methods

### 2.1. Selection of Suitable Linear B-Cell Epitope Predictors

The first priority of this work was to gather and test as many individual predictors as possible. However, the scope of methods that were to be tested could not be limitless, and thus some criteria for their selection were applied. At first, we decided to catalogue all available BCE predictors (Table 1). Out of all the catalogued predictors, about 18% were not available online, while the remaining 82% had corresponding website listed in the respective manuscript. However, this is when we first noticed an alarming trend; where many of the online tools of the predictors that we looked up were either offline for some hours during the day or—even worse—completely unreachable. As of the writing of this review, about 45% of the website reachable predictors are not currently available, most of which have been so for some time. Furthermore, even when operational, nine out of the eleven online servers have significant limitations on the number of sequences and the general workload they can process at a time. Considering the present issues and the future problems that might arise, we decided to resort only to methods that were available as standalone software, which became our main selection criterion. The second criterion was that methods should be usable via a CLI and not only through a Graphical User Interface (GUI) and the third criterion was that each method’s way of operation should be somewhat comparable and in tune with the rest of the available predictors. The main reason we limited ourselves to CLI tools was the technical limitation of the sheer volume of test sequences that had to be submitted to each predictor for our testing needs. Given that our test datasets contain tens of thousands of sequences, manual submission of those through a Graphical User Interface (GUI) becomes impossible. Out of the many methods that have been developed through the years (Table 1), seven were selected for testing: BcePred [31], BepiPred [33], ABCpred [34], COBEpro [19], SVMTriP [49], LBtope [51] and LBEEP [53]. Apart from the tools that were selected as part of our testing Epitopia was also available via CLI. Despite our efforts, Epitopia could not be locally installed because of dependency issues and thus has not been tested in this study. During our study, the second version of BepiPred was released, and its comparison with the rest of the methods and our decision not to utilize it in the development of the consensus method is discussed later in this article.

BcePred was published in 2004 by Raghava et al. [31], and is based on a plethora of physicochemical propensity scales utilizing amino acid properties, such as hydrophilicity and antigenicity, either individually or in combination. Moreover, it achieved a reported 56% sensitivity, 61% specificity and its highest accuracy of 58.70%, on a data set obtained from the database Bcipep [58], using a combination of flexibility, hydrophilicity, polarity and surface accessibility propensity scales.

BepiPred was developed in 2006 by Lund et al. [33], and it is the first ever method that utilizes an HMM. The HMM was trained using a data set derived from the database Antijen [59] and the Pellequer data set [27], and was then combined with Parker’s hydrophilicity scale, resulting in the BepiPred method. This method managed to achieve an Area Under Curve (AUC) of the Receiver Operating Characteristic (ROC) curve of 0.671 ± 0.013 on the Pellequer data set.

ABCpred was created in 2006 [34], again by the Raghava group and it was the first test case of a more sophisticated machine learning model. It is based on a Recurrent Neural Network (RNN) that was trained using a variety of different window sizes and hidden units. The window sizes that were tested, were 10, 12, 14, 16, 18, and 20. Thus six models were developed in total, with the window size of 16 amino acid residues achieving the highest accuracy of 65.93% and a Matthews Correlation Coefficient (MCC) of 0.3187, after fivefold cross-validation on a data set derived from Bcipep [58].

COBEpro was published in 2009 by Baldi et al. [19] at the University of California. This method utilizes a novel two-step system for the prediction of both linear and discontinuous B-cell epitopes. Firstly, it utilizes an SVM model to assign an epitopic propensity score to fragments within the given peptide sequence. Additionally, COBEpro is able to incorporate into the SVM model the provided or predicted secondary structure and solvent accessibility of the given sequence that are predicted by SSpro [60] and ACCpro [61], respectively. During the second stage, the method calculates an epitopic propensity score for each amino acid, based on the previous scores assigned by the model in the first stage. Among others, this predictor was tested on the fragmented version of Chen’s [39] data set, achieving an AUC of 0.829 and an accuracy of 78%.

SVMTriP was developed in 2012 [49] and it is an application of an SVM model that employs tri-peptide similarity calculated through the Blosum62 matrix in combination with amino acid propensity scales. Its prediction suite comes with six different models corresponding to window sizes of 10, 12, 14, 16, 18, and 20 of which the 20 amino acid residue model performed the best with a reported 80.10% sensitivity and 55.20% precision on a data set gathered from the Immune Epitope Data Base (IEDB) [62].

LBtope was the most recent effort, out of our selected predictors, on epitope prediction published by Raghava’s lab in 2013. This method uses, among other previously used types of features, a modified AAP profile from Chen’s method [39]. These profiles are used to convert the input sequence into numerical features that are then used as input for an SVM model that predicts epitopes. LBtope was trained and tested on a data set collected from IEDB, which comprised of experimentally verified epitopes and non-epitopes, in contrast to previous methods that used random peptides as non-epitopes. Its reported performance on different data sets varied significantly, with an accuracy ranging from 51.57% to 85.74%.

LBEEP was developed in 2015 by Saravan et al. [53] from the University of Madras in India. In this work, a novel amino acid feature descriptor called Dipeptide Deviation from Expected Mean (DDE) was developed, in an attempt to distinguish linear epitopes from non-epitopes. This new descriptor was then implemented with both SVM and AdaBoost-Random Forest machine learning techniques. The data set used to train this method was constructed by using only exact epitopes, instead of epitope containing regions, which have been used as training material in the past, making LBEEP a pioneer method in that respect. The exact epitopes used for training were isolated from IEDB [63] and are 5 to 15 amino acid residues long and thus LBEEP is better suited for predictions in that range. During testing, LBEEP achieved an accuracy between 61% and 73%, after fivefold cross-validation, on a data set derived also from IEDB.

Once all methods were installed in a local Unix-based machine, their output was validated by comparing example sequences of the local versions of software with the corresponding online tools. Additionally, all methods used in this analysis had their threshold set on its default value except for BcePred and COBEpro (Table 2). The default threshold values are set by the tools for a standard prediction but can also be found in their respective online tools when available, as well as the downloadable tool documentation. In the case of BcePred the default threshold value of the method used, which combines the results of four different propensity scales, was decreased from 2.38 to 2. This decrease was decided after extensive testing because the default threshold value proved to be extremely high. In essence, the higher threshold value of 2.38 reduced the method’s accuracy, while increasing its specificity, meaning the classifier was too selective when predicting for B-cell epitopes which led to a lot of potential epitopes being missed without an associated performance boost. Nevertheless, it should be noted that the new value used agreed with the default threshold currently used by both the online and the local version of the method, in contrast with the one reported in the initial publication. COBEpro on the other hand did not have a default threshold value since its results are printed out in a chart where epitopic propensity is given a relative positive or negative score for each position of the query protein. The threshold value that was chosen for this method was that of four positive votes above the baseline score of zero because it yielded the best results during testing.

### 2.2. Development of the Consensus Method

A consensus method was developed to incorporate all available methods that were selected in the first stage, and it is available upon request, due to free distribution limitations, at http://thalis.biol.uoa.gr/BCEconsensus/ (accessed on 21 March 2021) as a standalone application along with the source codes used for the testing and execution of our consensus classifier. The method was created using the PERL (Practical Extract Report Language) scripting language. All sequence-based methods can be divided into two categories based on their classification approach. The first category comprises of the methods that assign an epitopic propensity score to each residue of the provided sequence. Four methods are included in it: BcePred, BepiPred, COBEpro, and LBtope. The second category comprises of the methods that classify peptides within certain length sizes as epitopes or non-epitopes, such as ABCpred, SVMTriP, and LBEEP. The two categories are summarized in Table 3.

The methods that predict per peptide, ABCpred and SVMTriP, use predetermined fixed window sizes. Thus, it was necessary to choose a window size where these methods would operate sufficiently well, both in individual testing and as part of the consensus classifier. The window size chosen for these methods after initial testing was that of 20 residues. The main reasons were the better reported performance of SVMTriP at that window size and the lack of any default threshold values for the rest of the models in the documentation. As far as ABCpred is concerned, the performance penalty of selecting a window size of 20 instead of the reported best of 16 residues was minor. It should also be noted that initial testing for LBEEP at a window size of 20 was experimental, since the method was trained using only epitopes of lengths between 5 and 15, and thus any results outside that range were unreliable. Moreover, using the "confirmed model", as suggested by the creators of the method in the GitHub repository of LBEEP, returned worse results than the default "balanced" model, and so we opted to use the latter (Supplementary File S1/Table S1B).

Once a window size of 20 was selected for the “per peptide” methods, an effective strategy had to be formulated where the two different categories of output would produce a single consensus result. The solution was a consensus voting system that classifies a residue as belonging to an epitope when a predetermined threshold of votes has been achieved. When a “per residue” method classifies a residue of the query sequence as “epitopic” it counts as one positive vote, while when a “per peptide” method classifies a fragment of a protein as an epitope each amino acid of that peptide is classified as “epitopic”. So, when the sum of positive votes for a given position of a query sequence surpasses the threshold of the consensus classifier, the residue is marked as part of an epitope. The consensus threshold chosen, after testing, is defined as the hit overlap of at least half out of “n” selected methods, where “n” is the number of methods embedded in the algorithm [64]. The consensus method accepts protein sequences, of a length of 20 amino acid residues or higher, in FASTA format as input. The workflow of the consensus method is shown in (Supplementary File S1/Figure S2).

For testing purposes, a slightly different architecture of the consensus method was implemented, which specialized in rapid consensus output on our fixed length data sets. All methods—including the consensus—were mainly tested on a data set consisting of peptides with a length of 20. To resolve this issue, two parallel approaches were explored. In the first approach, all methods were included, and each method predicted whether an entire peptide is an epitope or not. However, in order for the results between the “per peptide” and “per residue” methods to be comparable, since only “per peptide” methods classify protein fragments, it was accepted that when “per residue” methods have predicted half or more of a peptide’s fragments as “epitopic”, then the whole peptide too is a predicted epitope. Such caveats are generally found in other forms of predictors of biological nature [65,66], and thus were chosen in our evaluation approach, as well. In the second approach, only “per residue” methods were included, and the consensus result was simply, a combination of only those predictions.

### 2.3. Data Sets

Typically, the development of machine learning classifiers requires both a training data set and a test data set, but since all the predictors tested in this work were previously developed, only the latter was necessary. However, due to the fact that the individual training data sets for each predictor contained a significant number of overlapping sequences, gathered from a select few databases (like IEDB [62] and Bcipep [58]), their inclusion in our test data set would introduce bias in the results. So, in order to test all the different methods in an unbiased manner, the positive and negative training data sets for each method were gathered from their respective publications and webpages. As shown in Table 4, the positive training data set for the majority of predictors comprises of all available BCEs from a given database, while the negative set contains random amino acid sequences from Swiss-Prot [67]. The way the negative set of control data is constructed, changed in algorithms developed after 2012 to include only sequences from confirmed non-epitopes, as is the case for SVMTriP, LBtope, and LBEEP. This change was introduced in order to improve the ability of prediction algorithms to effectively distinguish “epitopic” from random sequences, as it had been previously proposed [68].

While developing the consensus algorithm, a new version of BepiPred was published called BepiPred-2.0 [20]. Even though the method itself was not utilized in the development of the consensus method, its curated publicly available data set of linear epitopes was used as the source for this work’s data sets. This data set represents the biggest collection of linear epitope and non-epitope data used for the development of a prediction method to date, as IEDB is the largest and most frequently updated epitope database [63]. The BepiPred-2.0 data set was created by procuring from this database, all available epitopes (positive assay results) and non-epitopes (negative assay results), which were confirmed as such from two or more separate experiments. Afterwards, all peptides with a length smaller than 5 and longer than 25 residues were removed from the data set, because epitopes are rarely found outside this range [11]. Any epitopes that were found both in the positive and negative subsets were also removed. The resulting data set contains 11,834 epitopes in the positive subset and 18,722 non-epitopes in the negative subset. Aside from its curation, a useful feature of this data set was the mapping of all epitopes and non-epitopes on their respective parent protein sequence. This made extending each epitope to a desired length much easier.

The predictors that used IEDB as their source of epitope data are SVMTriP, LBtope, and LBEEP (Table 4). In order to produce an unbiased data set, their data sets were compared with BepiPred-2.0′s data set and all the matching peptides were removed. This resulted in our first data set, named Consensus_Redundant (Consensus_R) which comprises of 7675 epitopes and 15,617 non-epitopes. Using this data set as the source, a second non-redundant data set was constructed, by clustering peptides with the online tool CD-HIT [69]. All parameters were set to default and the sequence identity cut-off was set to 0.6 or 60%, as previously done in LBEEP’s data set creation [53]. The resulting data set was named Consensus_Non_Redundant (Consensus_NR) and it includes 4286 epitopes and 5266 non-epitopes. By creating the Consensus_NR data set in this manner, we essentially made the largest non-redundant data set possible, which contained known sequences that none of the predictors had previously “seen”. Additionally, from the Consensus_NR data set a subset was extracted, containing 552 epitopes and 480 non-epitopes with a peptide length of exactly 20 amino acids, which was named Consensus_NR_exact. This subset was used to test the performance of predictors using only true epitopes and not epitope containing regions that result from the extension-truncation technique. A summary of all data sets used in this study is presented in Table 5, while the complete data sets are provided in Supplementary Table S2.

Each data set used for testing contained peptides modified beforehand into fixed-length patterns using the technique of sequence extension and truncation, employed in previous methods [15,19,34,44]. This was done to accommodate the fixed-size input methods and thus included only their corresponding input lengths, namely 10, 12, 14, 16, 18, and 20 residues. For example, for a window size of 20, any epitopes or non-epitopes that were longer than 20 amino acids were shortened from both sides to have the desired length. Moreover, peptides with a length shorter than 20 residues were extended sideways on their parent sequence up to the desired length. The primary input size that was tested in this study was that of 20 residues for performance reasons as described in the development of the consensus algorithm. However, preliminary testing was also performed on a length of 16 residues, after analyzing the distribution of epitope lengths in the BepiPred-2.0 data set (Supplementary File S1/Figure S1). The mean peptide length of the data set was about 14 and the median value 15, which coincides with previous research on the characteristics of epitopes [11].

The workflow used to create the non-redundant data sets is shown in Supplementary File 1 (Supplementary File S1/Figure S3) and all data sets referenced in this section can be downloaded from this web page http://thalis.biol.uoa.gr/BCEconsensus/ (accessed on 21 March 2021).

### 2.4. Performance Measures

To evaluate a classifier’s performance both threshold dependent and independent metrics are used. The main threshold independent metric used in such cases is the AUC of the ROC curve. This metric was suggested as the preferred metric for benchmarking epitope prediction performance at a workshop by Greenbaum et al. [68], and thus it grew to become a standard in the BCE prediction field. However, because all the predictors that we examined were already fully developed and their optimal thresholds set, it did not make sense to use such a metric in our testing, since no model training was performed. For that reason, only threshold dependent metrics were employed, namely Sensitivity (SN), Specificity (SP), Accuracy (ACC), and Matthew’s Correlation Coefficient (MCC). Out of these metrics, significant attention was given to MCC, since it is generally regarded as the best performance metric for binary classifiers [70,71]. The coefficient’s value can range from −1 to +1, where the maximum value represents a perfect prediction and the minimum a total disagreement between predictions and observations. When the coefficient’s value is zero it indicates a prediction that is no better than random. Aside from the known value in accessing performance utilizing the MCC and accuracy metrics, regarding the other metrics, more importance was attached to sensitivity rather than specificity. Sensitivity indicates how effectively a predictive method manages to successfully locate areas that are actual epitopes, in contrast to specificity, which measures how effectively a predictive method manages to locate the sites that are not epitopes. In this study, the correctly predicted epitopes or “epitopic” residues were considered True Positive (TP), whereas the correctly predicted non-epitopes or “non-epitopic” residues were characterized as True Negative (TN). Conversely, the respective false predictions were defined as False Positive (FP) and False Negative (FN), respectively.

## 3. Results and Discussion

As mentioned in the Section 2, two approaches are followed to evaluate all predictions made by the consensus algorithm. In the first approach results from all methods are incorporated in the consensus method—both those predicting in a “per residue” and in a “per peptide” manner—while in the second approach the consensus prediction only utilizes the “per residue” methods. Two different versions of the consensus algorithm were created in the “per peptide” mode, as seen in Table 6; one which includes all predictors and one which utilizes all of them except LBEEP. This was done after noticing that LBEEP performs much worse, compared to the rest of the predictors. This performance issue can be mainly attributed to the fact that the optimal prediction window of 5–15 residues for LBEEP is different than the 20-residue length that was used for our testing purposes (Table 3).

The evaluation of the predictors’ performance was done primarily by measuring their MCC values, while secondary importance was assigned to achieving higher accuracy, and sensitivity. Sensitivity was considered more important than specificity for this particular application since a BCE predictor’s primary goal is to find possible BCEs in unknown sequences. Naturally, sensitivity and specificity are not mutually exclusive entities, yet in this study optimal sensitivity is preferred to optimal specificity. For further testing results please refer to Supplementary File S1.

### 3.1. Performance of All Predictors on Consensus_NR

The results regarding the “per peptide” approach (Table 6) show that the highest MCC value was achieved by the BepiPred method with 0.0778, followed by our Consensus_NoLBEEP algorithm—the one without LBEEP—that achieved an MCC of 0.0721. Moreover, LBEEP had the lowest MCC (-0.0103), while BcePred and SVMTriP also scored low (0.0251 and 0.0290, respectively). The highest accuracy was achieved by our Consensus_ALL method with 55.59%, which was marginally better than those of SVMTriP and BcePred. SVMTriP had the best specificity out of all the methods (85.87%), followed by LBEEP and BcePred. Additionally, the ABCpred method achieved the greatest sensitivity with 66.44%, and COBEpro achieved the second highest with 58.63%. The Consensus_NoLBEEP algorithm achieved values close to the best for both MCC and accuracy, and also had a relatively improved MCC and a significantly increased sensitivity compared to its first version.

In the case of the “per residue” approach (Table 7), the consensus method (Consensus_RES) achieved the best MCC with 0.489, while BepiPred scored marginally worse with 0.0488. The same pattern was also observed for accuracy, where the Consensus_RES method scored 53.04% and BepiPred 52.88%. The greatest sensitivity was achieved by COBEpro with 49.27%, while BepiPred was again second best with 48.12%. The worst performance regarding MCC was attained by BcePred and COBEpro with scores of 0.0154 and 0.0175, respectively. Overall, despite the slight improvement in MCC and accuracy, the performance of the consensus algorithm was not significantly better in any of the statistical measures examined in the second part of the results.

When comparing the results of the two approaches only minor differences in performance are observed between the two modes of prediction for the four “per residue” methods. Generally, we notice a slight decrease in MCC from a maximum of 0.0778 in the first approach to a maximum of 0.0489 in the second, while accuracy is comparatively worse on average. Out of the “per residue” methods, BepiPred comes on top in both approaches in MCC and accuracy. The Bcepred method appears to perform relatively worse than the rest in both groups with the lowest MCC in both cases, whereas the COBEpro method performs relatively better in its “per peptide” iteration, with an average MCC score in the first part but a poor score in the second segment of the results. Moreover, in both approaches, our consensus algorithm does not significantly outperform the rest of the predictors and only achieves a performance that is quite similar to that of BepiPred.

In summary, we observe that in all cases: MCC values are less than 0.1, accuracy is ranging from 50% to 55%, there are relatively high specificity values in certain cases such as SVMTriP and BcePred, and sensitivity values are low. Aside from our consensus methods, the best performers were LBtope and BepiPred and the worst ABCpred and LBEEP, which also displayed the lowest MCC scores.

Using the Consensus_NR data set we implemented many iterations of the consensus method utilizing many different method combinations, in order to find the optimum. As expected, LBEEP’s presence undermined the consensus predictor’s performance, and it was therefore omitted from the final version (Consensus_NoLBEEP) and any further testing in the 20-residue window size. It was also observed that ABCpred overestimated the presence of epitopes in their respective peptides, which led to reduced accuracy and increased sensitivity. Nevertheless, it remained part of the final consensus algorithm to improve its overall sensitivity.

At this point, it should be noted that LBEEP was also tested on a peptide length of 14-residues since the method was reported to perform optimally when a window size between 5 and 15 residues is used for prediction. Results showed that the method indeed performs better at this window size, but it is still marginally better than a random prediction according to its MCC (Supplementary File S1/Table S1A). Even though, the results were better for LBEEP the rest of the methods either cannot be used at that window size or perform way worse than what we had already seen and so we opted to not use the 14-residue window any further.

### 3.2. Overall Method Performance and Comparison with BepiPred-2.0

**Figure 1:**
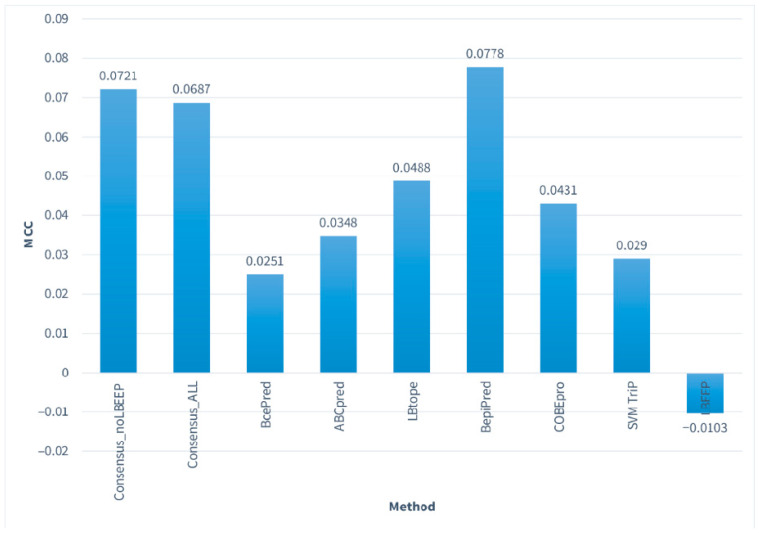
Matthews Correlation Coefficient (MCC) values achieved by all methods tested on the Consensus_NR data set at 20 amino acid residues in “per peptide” mode. The vertical axis represents the MCC value for all the methods and the horizontal axis the names of these methods. The best MCC is achieved by the BepiPred method, followed closely by our Consensus methods, while the worst performers are the LBEEP, SVMTriP, and BcePred methods.

The performance of the linear B-cell epitope predictors examined was found to be poor in the data sets and window sizes used during testing (Figure 1).

Additionally, despite our optimization, our consensus method performed only marginally better than the rest of the methods, thus nullifying its usefulness. We believe that the problems which may explain these results can be divided into two categories: those concerning the individual methods and those of the consensus approach.

The first problem regarding the prediction methods is that the epitope data used to train and test them, and as a result, the methods themselves are outdated. This probably is what caused their significantly reduced performance in our contemporary and considerably larger set of data. Furthermore, the general difficulty of creating a relatively reliable sequence-based predictor is well known, in contrast with those available for example in the prognosis of T-cell epitopes [72]. This is mainly due to the 3D nature of all B-cell epitopes, which consist of seemingly unrelated residue patterns of the antigen. Their emergence is also subject to multiple factors, such as antigen concentration and the type of chemical test [68].

In our attempt to create a consensus predictor, the first problem we encountered was the different modes of operation of the individual prediction methods, namely their distinction into “per peptide” and “per residue” predictors. To effectively compare the two modes, “per residue” predictor outputs were converted to “per peptide”, by using a percentage cut-off to classify peptides as epitopes and non-epitopes. This, however, is not their intended operation mode, which certainly influences the performance of these methods and thus the performance of the consensus method.

Another obstacle in this effort was time and complexity. The prediction and evaluation process for all possible windows (10, 12, 14, 16, 18, and 20) is very time-consuming. This also had to be performed for as many predictors as possible to make the consensus classifier more effective, leading to a significant increase in software development complexity as the number of incorporated predictors grew. In addition, accurate assessment of the viability of such an effort is very difficult, due to the inability to accurately compare them beforehand using the results presented in the corresponding publications, as there is no single set of evaluation data or metrics [15]. Finally, there was a lack of variety in the methods utilized in our selected predictors, where most of them were based on SVM models, which may have negatively affected the performance of our consensus predictor [73].

When comparing all of the methods we tested, with some of the newer methods such as BepiPred-2.0 and iBCE-EL, which were tested on large non-redundant data sets much like the ones we used, their reported superiority is apparent. Out of the two, BepiPred-2.0 was released during the initial part of testing in our research, and as such, it was a likely candidate for our consensus method. However, after observing the poor performance of all the different methods tested against its data set, we decided to not include it in our consensus approach, but simply to use it as a reference for what a modern predictor can achieve versus the older ones. Unlike its predecessor, BepiPred-1.0, and most other sequence-based predictors, BepiPred-2.0 is trained exclusively on epitope data derived from antigen–antibody crystal structure complexes obtained from the Protein Data Bank [74]. This was done in order to combat the generally poor performance of predictors, which can be partly attributed to poorly annotated and noisy training data, in comparison with data derived from crystal structures which is presumed to be of higher quality and indeed resulted in a significantly improved predictive power [20]. From these complexes, all antigenic residues close enough to their respective antibody were gathered. These residues became the positive subset of the training data set, while the negative subset was constructed from randomly selected non-epitope residues.

While BepiPred-2.0 was trained using epitope data derived only from 3D structures, its performance on linear BCEs was also benchmarked on one such data set. We compared the performance of BepiPred-2.0 against our Consensus_noLBEEP predictor using the Consensus_NR dataset at a window size of 20 amino acid residues. When compared to our consensus method, BepiPred-2.0 has a similar performance in accuracy and MCC, but exhibits higher sensitivity and lower specificity, as shown in the comparison performed in Table 8. However, the results for both methods are far from optimal, and a lot of work still remains to be done in order to create a predictor that will perform optimally during linear BCE detection.

## 4. Conclusions

In summary, in this paper, we independently evaluated the performance of seven of the most popular linear B-cell epitope predictors on the largest unbiased data set possible. In the process, we also presented the course of design, development, and evaluation of a consensus prediction algorithm for linear B-cell epitopes. The performance of all predictors, except for LBEEP on whom testing was exploratory, was found marginally better than random classification. Additionally, our Consensus classifier failed to significantly outperform its constituent methods. While the method comparison was performed with some necessary compromises, we believe that this update in performance can help to better inform researchers that wish to consult some of these tools to facilitate and direct their research. Instead, we should also like to suggest that researchers opt for some of the newer predictors referenced in this work, like BepiPred-2.0.

An excellent and timely example of the significance of using well-performing systems for the prediction of BCEs—in the context of the ongoing global pandemic—are studies aiming at the in silico multi-epitope vaccine design for SARS-CoV-2 and other antigenic systems [75,76,77]. For the studies that have been performed up to now, a holistic approach is adopted where not only linear BCEs are predicted for the antigenic system of interest but several other characteristics leading to suitable vaccine candidate such as: the presence of conformational BCEs, T-cell epitopes, and the antigenicity of the predicted peptides. This is a more realistic approach for predicting a vaccine candidate since the presence of linear BCEs alone can hardly elicit the immune response necessary for successful vaccination. However, most studies employ some of the same problematic linear BCE predictors that have been reviewed herein, which could result in unsatisfactory results as far as linear BCEs are concerned. In a recent study, researchers developed DeepVacPred [76], which is a deep learning framework and as part of their framework included BepiPred-2.0, SVMtrip, ABCPred, and BCPREDS for linear BCE prediction. Instead of using them all together they first used BepiPred-2.0 to find epitopes on the SARS-CoV-2 spike protein and then used the other three predictors to validate the results of BepiPred-2.0, in essence using multiple predictors to “sift” their results. Perhaps, such an approach is appropriate for the current state of B-cell epitope predictors until further progress has occurred, however it only goes to show that more work needs to be done on the field, and benchmarking of available methods, like the one we have done in this work, can only aid towards the choice of appropriate methods for ensemble classifiers.

Finally, due to the apparent difficulty of constructing an accurate general-purpose linear BCE predictor, we would like to stress the importance of focusing software development on the creation of more specialized predictors for specific antigenic systems, such as known viruses or viral families of high interest. These methods could be employed when new viruses of the same family arise, like SARS-CoV-2 arose out of the family of Coronaviridae. In turn, this could lead to optimization in the feature selection process during classifier training and better predictive performance within that limited scope, which would prove very important towards the development of better vaccines in the future.

## Figures and Tables

**Table 1:** Linear B-cell epitope predictors in chronological order, alongside a short description of their methodology, their current status and their web page. After researching the relevant publications, we gathered up all the linear B-cell epitopes predictors we could find in this fairly complete, but not exhaustive catalogue. For every method we reference the source material to determine their methodology, which we have summed up for each predictor in a short description. For every predictor we also checked their availability status, as of writing this review, and categorized them regarding their general and current availability online as tools, as well as their obtainability as standalone software packages. We also provide the institution in which they were developed. In the last column, we provide the website links for each method, when available.

Predictor	Description	Status	Institution	Link
**Antigenic** [24]	Physico-chemical propensity scales, occurrence of residues	Not currently available online	Department of Zoology, University of Poona, India	http://www.emboss.bioinformatics.nl/cgi-bin/emboss/antigenic
**PEOPLE** [29]	Physico-chemical propensity scales	Not available online	Laboratoire de Spectroscopies et Structures Biomoléculaire, Université de Reims Champagne Ardenne, France	-
**BEPITOPE** [30]	Physico-chemical propensity scales	Freely available online	Ιnstitute of environmental biology and biotechnology, CEA, France	http://bepitope.ibs.fr/
**BcePred** [31]	Physico-chemical propensity scales	Freely available online and downloadable	Ιnstitute of Microbial Technology, Chandigarh, Ιndia	http://crdd.osdd.net/raghava/bcepred/index.html
**BepiPred-1.0** [33]	HMM & Parker hydrophilicity scale	Freely available online and downloadable	Center for Biological Sequence Analysis, Technical University of Denmark	http://www.cbs.dtu.dk/services/BepiPred-1.0/
**Söllner** [47]	Physicochemical Properties, Molecular Operating Environment, K-Nearest Neighbor, Decision Tree	Not available online	emergentec biodevelopment GmbH, Vienna, Austria	-
**Chen** [39]	SVM & AAP	Not available online	Institute of Image Processing and Pattern Recognition, Shanghai Jiaotong University, Shanghai, China	-
**ABCpred** [34]	Neural networks (feed forward & reccurent)	Freely available online and downloadable	Ιnstitute of Microbial Technology, Chandigarh, Ιndia	http://crdd.osdd.net/raghava/abcpred/index.html
**BCPREDS** [36,37]	SVM	Freely available online and downloadable	Dep. of Computer Science & Dep. of Genetics, Development and Cell Biology, Ιowa State University, USA	http://ailab.ist.psu.edu/bcpred/
**AAPPred** [38]	SVM & AAP	Not currently available online	Faculty of Biology, Moscow State University Russia	http://www.bioinf.ru/aappred/predict
**Epitopia** [35]	Machine Learning algorithm trained to discern antigenic features	Freely available online and downloadable	Tel Aviv Uni. Ιsrael & Uni. of British Columbia Canada & Uni. of Massachusetts, USA	http://epitopia.tau.ac.il/index.html
**COBEpro** [19]	SVM	Freely available online and downloadable upon request	Dep. of Computer Science and Ιnstitute for Genomics and Bioinformatics, University of California USA	http://scratch.proteomics.ics.uci.edu/
**BayesB** [40]	SVM	Not currently available online	Singapore Ιmmunology Network & Dep. of Biochemistry, National Uni. of Singapore	http://immunopred.org/bayesb/index.html
**LEPS** [41]	SVM & Physicochemical propensity scales & Amino Acid Segments	Not currently available online	National Taiwan Ocean University Taiwan, China Medical University Taiwan	http://leps.cs.ntou.edu.tw/
**BEOracle** [42]	SVM	Not available online	Department of Biostatistics and Computational Biology, Dana-Farber Cancer Ιnstitute & Harvard School of Public Health, Boston, USA	-
**BEST** [48]	SVM	Not currently available online	School of Mathematical Sciences and LPMC, Nankai University, Tianjin, People’s Republic of China	http://biomine.ece.ualberta/
**SVMTriP** [49]	SVM	Freely available online and downloadable	University of Nebraska USA, Osaka Uni. Japan	http://sysbio.unl.edu/SVMTriP/
**BEEPro** [50]	SVM & Physicochemical propensity scales & Position Specific Scoring Matrix	Not available online	School of Medicine, Taipei Medical University, Taipei, Taiwan	-
**LBtope** [51]	SVM & Physicochemical propensity scales & AAP	Freely available online and downloadable	Ιnstitute of Microbial Technology, Chandigarh, Ιndia	http://crdd.osdd.net/raghava/lbtope/protein.php
**Random Forest** [52]	Amino acid descriptors & Random Forest	Not currently available online	Research Center of Modernization of Traditional Chinese Medicines, Central South University, Changsha, China	http://sysbio.yznu.cn/Research/Epitopesprediction.aspx
**EPMLR** [43]	Multiple Linear Regression	Not currently available online	The Key Laboratory of Bioinformatics, Ministry of Education, School of Life Sciences, Tsinghua University, Beijing, China	http://www.bioinfo.tsinghua.edu.cn/epitope/EPMLR/
**DMN-LBE** [44]	Deep Maxout Networks	Not currently available online	The Key Laboratory of Bioinformatics, Ministry of Education, School of Life Sciences, Tsinghua University, Beijing, China	http://bioinfo.tsinghua.edu.cn/epitope/DMNLBE/
**LBEEP** [53]	Deviation from Expected Mean—SVM	Freely available download	Center for Advanced Study in Crystallography and Biophysics, University of Madras, Guindy Campus, Chennai, Tamil Nadu, Ιndia.	https://github.com/brsaran/LBEEP
**APCpred** [54]	Amino acid Anchoring Pair Composition & SVM	Not currently available online	Department of Molecular Biology, Hebei University College of Life Sciences, China	http://ccb.bmi.ac.cn/APCpred/
**DRREP** [45]	Deep Ridge Neural Network	Not currently available online	Department of Computer Science, University of Central Florida, Orlando, FL, USA	https://github.com/gsher1/DRREP
**BepiPred-2.0** [20]	Random forest algorithm trained on epitopes derived from crystal structures	Freely available online and downloadable	Department of Bio and Health Informatics, Technical University of Denmark, Denmark	http://www.cbs.dtu.dk/services/BepiPred/
**iBCE-EL** [46]	Ensemble framework combining ERT & GB	Freely available online	Department of Physiology, Ajou University School of Medicine, Suwon, South Korea	http://thegleelab.org/iBCE-EL/

HMM: Hidden Markov Model, SVM: Support Vector Machine, AAP: Amino Acid Pairs, ERT: Extremely Randomized Tree, GB: Gradient Boosting, CEA: Commissariat à l’énergie atomique et aux énergies alternatives.

**Table 2:** A summary of methods, threshold values, and modifications applied to each predictor. Each predictor first had its best performing mode selected and its threshold value set to a specific value shown in the table, using the criteria described in the manuscript.

Predictor	Threshold	Mode	Threshold Type
BcePred	2	Combined	Not Default
BepiPred-1.0	0.35	BepiPred	Default
ABCpred	0.51	20	Default
COBEpro	4	-	Not Default
SVMTriP	0.2	20	Default
LBtope	0.6	LBtop_Confirm	Default
LBEEP	0.6	Balanced	Default

**Table 3:** Input window sizes and prediction approach of each method. The classification of query proteins as epitopes can generally be performed in either a “per residue” or a “per peptide” basis. In the “per residue” methods each separate residue of a protein is assigned an antigenicity score, while in the “per peptide” methods, a prediction is limited within fixed windows sizes.

Predictor	Prediction	Window Size
ABCpred	Per peptide	10, 12, 14, 16, 18, 20
SVMTriP	Per peptide	10, 12, 14, 16, 18, 20
LBEEP	Per peptide	5–15
BcePred	Per residue	-
BepiPred-1.0	Per residue	-
COBEpro	Per residue	-
LBtope	Per residue	-

**Table 4:** A summary of the source of positive and negative data sets for each predictor. For every predictor, a database had to be used to construct its training data sets, which comprise of a positive and a negative subset of data. In this table, we outline the database or curated data set from which each method sourced its training data set, along with the date that the data was obtained. The date could be used to determine the snapshot of the data, which could have been obtained for each predictor’s training, allowing us to determine possible overlaps of our testing data set with the relevant training data.

Predictor	Positive	Negative
BcePred	BCIPEP (2004)	1029 random sequences
BepiPred-1.0	HΙV/PELLEQUER/ANTIJEN	Not described in the original publication
ABCpred	BCIPEP (2006)	700 random sequences
COBEpro	HΙV/PELLEQUER	HIV/Pellequer non-Epitopes
SVMTriP	ΙEDB (2012)	4925 IEDB non-epitopes
LBtope	ΙEDB (2012)	IEDB (2012) non-epitopes
LBEEP	ΙEDB (2015)	IEDB (2015) non-epitopes

**Table 5:** A summary of test data sets utilized in this study. The counts of positive and negative subsets of data used in each of the three data sets developed for method testing is shown.

Data Set	Epitopes	Non-Epitopes
BepiPred-2.0 *	11,814	18,689
Consensus_R	7675	15,617
Consensus_NR	4286	5266

* A slightly modified version of BepiPred-2.0′s data set was used, which had a few epitopes removed because their sequence of origin was shorter than 20 amino acid residues, and thus the epitope could not be extended to the desired length.

**Table 6:** Performance of all predictors in “per peptide” mode. The methods are tested against the Consensus_NR (Non_Redundant) data set.

Predictor	SN%	SP%	ACC%	MCC
Consensus_noLBEEP	48.39	58.81	54.14	**0.0721**
Consensus_ALL	27.15	78.73	55.59	0.0687
BcePred	22.21	79.85	53.99	0.0251
ABCpred	66.44	36.9	50.16	0.0348
LBtope	45.91	58.94	53.1	0.0488
BepiPred-1.0	49.95	57.84	54.3	**0.0778**
COBEpro	58.63	45.67	51.49	0.0431
SVMTriP	16.21	85.87	54.62	0.0290
LBEEP	19.06	80.12	52.72	−0.0103

SN: Sensitivity, SP: Specificity, ACC: Accuracy, MCC: Matthew’s Correlation Coefficient.

**Table 7:** Performance of “per residue” predictors. The methods are tested against the Consensus_NR data set.

Predictor	SN%	SP%	ACC%	MCC
Consensus_RES	46.64	58.24	53.04	0.0489
BcePred	29.18	72.21	52.9	0.0154
LBtope	45.56	57.47	52.13	0.0304
BepiPred-1.0	48.12	56.76	52.88	0.0488
COBEpro	49.27	52.49	51.05	0.0175

SN: Sensitivity, SP: Specificity, ACC: Accuracy, MCC: Matthew’s Correlation Coefficient.

**Table 8:** Comparison of the performance of our consensus predictor and BepiPred-2.0 against the Consensus_NR data set.

Predictor	SN%	SP%	ACC%	MCC
Consensus_noLBEEP	50.18	58.54	54.07	0.0873
BepiPred-2.0	63.35	42.63	51.93	0.0607

SN: Sensitivity, SP: Specificity, ACC: Accuracy, MCC: Matthew’s Correlation Coefficient.

## Data Availability

All data used in this study are available at http://thalis.biol.uoa.gr/BCEconsensus/ (accessed on 21 March 2021).

## Supplementary Materials

The following are available online at https://www.mdpi.com/1422-0067/22/6/3210/s1. Table S1 (File S1): (A) LBEEP’s performance on peptides of 14-amino acid residues. (B) LBEEP’s performance using 3 different models for the 20 amino acid residue peptides of the Consensus_NR data set. Table S2: Evaluation Datasets. File S1: Figure S1: Distribution of epitope lengths in the Bepipred 2.0 data set. Figure S2 showing the workflow of the consensus method and Figure S3 showing the workflow of the creation of the non-redundant data sets. Supplementary results and discussion and Tables S3–S6.

## Abbreviations

BCE	Linear B-cell Epitope
HMM	Hidden Markov Model
SVM	Support Vector Machine
AAP	Amino Acid Pairs
ERT	Extremely Randomized Tree
GB	Gradient Boosting
CLI	Command Line Interface
GUI	Graphical User Interface
AUC	Area Under Curve
ROC	Receiver Operating Characteristic
MCC	Matthews Correlation Coefficient
IEDB	Immune Epitope Data Base
Consensus_R	Consensus_Redundant
Consensus_NR	Consensus_Non_Redundant
SN	Sensitivity
SP	Specificity
ACC	Accuracy
TP	True Positive
TN	True Negative
FP	False Positive
FN	False Negative
